# Me-LLaMA: Medical Foundation Large Language Models for Comprehensive Text Analysis and Beyond

**DOI:** 10.21203/rs.3.rs-5456223/v1

**Published:** 2024-12-18

**Authors:** Qianqian Xie, Qingyu Chen, Aokun Chen, Cheng Peng, Yan Hu, Fongci Lin, Xueqing Peng, Jimin Huang, Jeffrey Zhang, Vipina Keloth, Xinyu Zhou, Lingfei Qian, Huan He, Dennis Shung, Lucila Ohno-Machado, Yonghui Wu, Hua Xu, Jiang Bian

**Affiliations:** Yale University; Yale University; University of Florida; University of Florida; University of Texas Health Science, Center at Houston; Yale University; Yale University; Yale University; Yale University; Yale University; Yale University; Yale University; Yale University; Yale University; Yale University; University of Florida; Yale University; University of Florida

## Abstract

Recent advancements in large language models (LLMs) like ChatGPT and LLaMA have shown significant potential in medical applications, but their effectiveness is limited by a lack of specialized medical knowledge due to general-domain training. In this study, we developed Me-LLaMA, a new family of open-source medical LLMs that uniquely integrate extensive domain-specific knowledge with robust instruction-following capabilities. Me-LLaMA comprises foundation models (Me-LLaMA 13B and 70B) and their chat-enhanced versions, developed through comprehensive continual pretraining and instruction tuning of LLaMA2 models using both biomedical literature and clinical notes. Me-LLaMA utilized the largest and most comprehensive medical data, including 129B pre-training tokens and 214K instruction tuning samples from diverse biomedical and clinical data sources. Training the 70B models required substantial computational resources, exceeding 100,000 A100 GPU hours. We applied Me-LLaMA to six medical text analysis tasks and evaluated its performance on 12 benchmark datasets. To further assess Me-LLaMA’s potential clinical utility, we evaluated its performance on complex clinical case diagnosis compared with other commercial LLMs, using both automatic and human evaluations. Me-LLaMA models outperform LLaMA, and other existing open-source medical LLMs in both zero-shot and supervised learning settings for most text analysis tasks. With task-specific instruction tuning, Me-LLaMA models also surpass leading commercial LLMs, outperforming ChatGPT on 7 out of 8 datasets and GPT-4 on 5 out of 8 datasets. Moreover, Me-LLaMA’s performance is comparable to ChatGPT and GPT-4 for diagnosing complex clinical cases. Our findings underscore combining domain-specific continual pretraining with instruction tuning is essential for developing effective domain-specific large language models in healthcare, significantly enhancing performance across diverse medical text analysis tasks and applications. By publicly releasing our models and resources under appropriate user agreements, we aim to foster innovation and facilitate advancements in medical AI, benefiting researchers and practitioners within the community.

## INTRODUCTION

Large language models (LLMs) have shown great potential in improving medical applications such as clinical documentation, diagnostic accuracy, and patient care management.^1,2,3^ However, general-domain LLMs often lack specialized medical knowledge because they are primarily trained on non-medical datasets^4^, limiting their effectiveness in healthcare settings. Although commercial LLMs, such as ChatGPT ^5^ and GPT-4,^6^ offer advanced capabilities, their closed-source nature restricts the flexible customization and accessibility required for medical use. This limitation has spurred the research towards developing open-source LLMs such as LLaMA;^7–8^ Yet, these models still fall short due to their general-domain training.^9–10^

To address these challenges, researchers have explored strategies to develop domain specific LLMs for the medical domain. Instruction fine-tuning of general-domain models, as seen in MedAlpaca,³ ChatDoctor,¹² and AlpaCare,¹³ attempts to enhance medical capabilities but is limited by the base models’ lack of specialized knowledge; instruction fine-tuning alone cannot compensate for this deficiency. Training models from scratch using medical corpora, exemplified by GatorTronGPT,¹ overcomes this limitation but demands substantial computational resources and time. A more cost-effective alternative is continual pretraining, enabling models to acquire specialized medical knowledge while leveraging existing model architectures; notable examples include PMC-LLaMA,¹ Meditron¹ and Clinical LLaMA.¹

Despite these advances, existing LLMs of continual pretraining in the medical domain exhibit notable limitations: (1) Although both domain knowledge and instruction-following capabilities are crucial, only PMC-LLaMA¹ has combined continual pretraining with instruction fine-tuning, revealing a gap in leveraging the synergy between these two aspects. (2) Only one model (Clinical LLaMA) used clinical notes from electronic health records, which is crucial for real-world clinical applications as it provides context-specific information from direct patient care. None of the existing models used both biomedical literature and clinical notes, which is one of the goals of this project. (3) Due to the limited medical datasets utilized for model development, these models still lack essential domain knowledge, which hampers their effectiveness. By combining biomedical literature and clinical notes, we generated the largest biomedical pre-training dataset (129B tokens), compared to the previous efforts (i.e., 79B tokens in PMC-LLaMA as the highest, see Table 1). (4) Evaluations have predominantly centered on medical question-answering (QA) tasks, lacking comprehensive assessments on the generalizability of those foundation models across diverse medical tasks.

To overcome these limitations, we present Me-LLaMA, a novel family of open-source medical large language models that uniquely integrate extensive domain-specific knowledge with robust instruction-following capabilities. Me-LLaMA comprises foundation models (Me-LLaMA 13B and 70B) and their chat-enhanced versions, developed through comprehensive continual pretraining and instruction tuning of LLaMA2 models. Leveraging the largest and most diverse medical dataset to date—combining 129 billion pretraining tokens and 214,000 instruction samples from scientific literature, clinical guidelines, and electronic health record clinical notes—Me-LLaMA excels across a wide spectrum of medical text analysis and real-world clinical tasks. Unlike prior studies, we conduct the most extensive evaluation to date, covering six critical tasks—question answering, relation extraction, named entity recognition, text classification, text summarization, and natural language inference—across twelve datasets from both biomedical and clinical domains. Our results demonstrate that Me-LLaMA not only surpasses existing open-source medical LLMs in both zero-shot and supervised settings but also, with task-specific instruction tuning, outperforms leading commercial LLMs such as ChatGPT on seven out of eight datasets and GPT-4 on five out of eight datasets. Furthermore, to evaluate Me-LLaMA’s potential clinical utility, we assessed the models on complex clinical case diagnosis tasks, comparing their performance with other commercial LLMs using both automatic and human evaluations. Our findings indicate that Me-LLaMA’s performance is comparable to that of ChatGPT and GPT-4, despite their substantially larger model sizes.

Our findings underscore the importance of combining domain-specific continual pretraining with instruction tuning to develop effective large language models for the medical domain. Recognizing the significant resources required, we have publicly released our Me-LLaMA models on PhysioNet under appropriate Data Use Agreements (DUAs) to lower barriers and foster innovation within the medical AI community. Alongside the models, we provide benchmarks and evaluation scripts on GitHub to facilitate further development. We anticipate that these contributions will benefit researchers and practitioners alike, advancing this critical field toward more effective and accessible medical AI applications.

## METHODS

**Figure 1:**
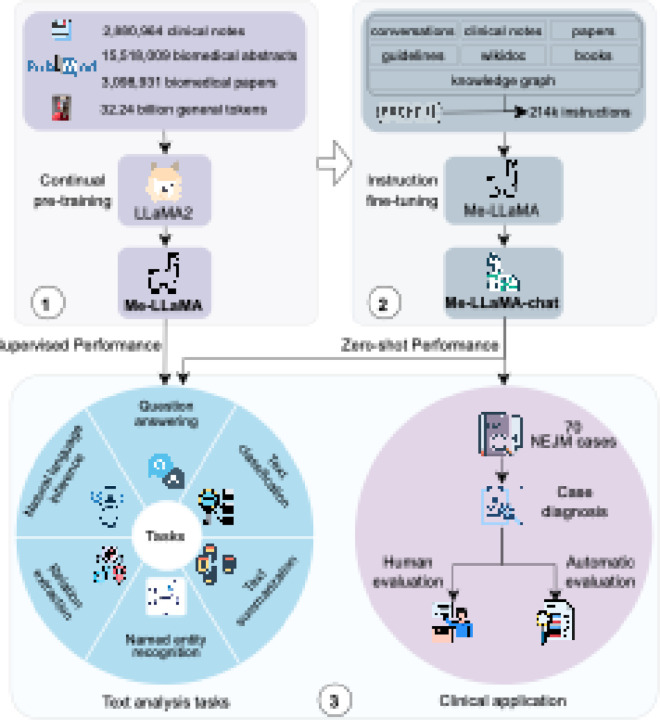
Overview of the study. Our study has three main components including pre-training, instruction fine-tuning and evaluation. Pre-training: we firstly developed the Me-LLaMA base models by continual pre-training LLaMA2 with 129 billion tokens from mixed pre-training text data. Instruction fine-tuning: Me-LLaMA-chat models were further developed by instruction tuning Me-LLaMA base models with 214K instructions. Evaluation: Finally, we evaluated the Me-LLaMA base models in a supervised learning setting across six text analysis tasks, and the Me-LLaMA-chat models in a zero-shot setting on both text analysis tasks and a clinical diagnosis task.

We utilized LLaMA2 as the backbone model and developed Me-LLaMA through the process of continual pre-training and instruction tuning of LLaMA2, using 129B tokens and 214K instruction tuning samples from general, biomedical, and clinical domains. Figure 1 shows an overview of our study.

### Continual Pre-Training Data

To effectively adapt backbone LLaMA2 models for the medical domain through continual pre-training, we developed a mixed continual pre-training dataset, comprised of biomedical literature, clinical notes, and general domain data. It integrates over 3 million full biomedical articles from PubMed Central and over 15 million paper abstracts from PubMed, sourced from the Pile dataset.^14^ To incorporate real-world clinical scenarios and reasoning, we included de-identified free-text clinical notes from MIMIC-III,^15^ MIMIC-IV,^16^ and MIMIC-CXR.^17^ Moreover, to avoid the model forgetting acquired general knowledge, we incorporated a subset from the RedPajama^18^ dataset, a replication of LLaMA2’s pre-training data. The dataset was structured with a 15:1:4 ratio of biomedical, clinical, to general domain data and contains a total of 129 billion tokens, making it the largest pre-training dataset in the medical domain currently available.

### Medical Instruction Tuning Data

To enhance our model’s ability to follow instructions and generalize across diverse medical tasks, we further developed a novel medical instruction tuning dataset with 214,595 high-quality samples from a wide array of data sources. This dataset stands out from those used in existing medical LLMs due to its comprehensive coverage of both biomedical and clinical domains. Our data sources included biomedical literature, clinical notes, clinical guidelines, wikidoc, knowledge graphs, and general domain data, as shown in Table 2. The diverse tasks aim to refine the model’s ability to process and respond to medical information accurately and contextually. Detailed prompts for each data and the data example are shown in Appendix 0.1, Table A.1.

### Training Detatils

As shown in Fig. 3, we developed the Me-LLaMA 13B and 70B base models by continual pre-training the LLaMA2 13B and 70B models. These base models were then instruction-tuned to create the Me-LLaMA-13B-chat and Me-LLaMA-70B-chat models.

#### Me-LLaMA base models – continual pretraing LLaMA2

This phase aims to adapt LLaMA2 models to better understand and generate text relevant to the medical context using the pre-training datasets we constructed. The training involves sequences of medical texts, where the model learned to predict the next token in a sequence, maximizing the likelihood, where is the parameter set of LLaMA2 models. This training was executed on the University of Florida’s HiPerGator AI supercomputer with 160 A100 80GB GPUs. We employed the AdamW optimizer with hyperparameters set to to 0.9 and to 0.95, alongside a weight decay of 0.00001 and a learning rate of 8e-6. We used a cosine learning rate scheduler with a 0.05 warmup ratio for gradual adaptation to training complexity and bf16 precision for computational efficiency. Gradient accumulation was set to 16 steps, and training was limited to one epoch. We utilized DeepSpeed^26^ for model parallelism.

#### Me-LLaMA chat models – instruction fine-tuning ME-LLaMA:

We further fine-tuned Me-LLaMA base models, using the developed 214k instruction samples. The training objective is to maximize the likelihood:

$𝓛\left(\text{Θ}\right)=argmax\sum_{\left(x^{i},y^{i}\right)\in \left(X,Y\right)}\log p\left(y^{i}|x^{i};\text{Θ}\right)$, where $x^{i}$ represents the input instruction, $y^{i}$ is the ground truth response, and Θ is the parameter set of Me-LLaMA. Executed using 8 A100 GPUs, the fine-tuning process was set to run for 3 epochs with a learning rate of 1e-5. We used a weight decay of 0.00001 and a warmup ratio of 0.01 for regularization and gradual learning rate increase. We utilized LoRA-based^27^ parameter-efficient fine-tuning.

### Evaluation Benchmark

#### Biomedical and clinical NLP tasks:

Existing studies^2,3,10,11^ in the medical domain have primarily focused on evaluating the QA task. In this study, we build an extensive medical evaluation benchmark (MIBE), encompassing six critical text analysis tasks: QA, NER, RE, Text Classification, Text Summarization and NLI. These tasks collectively involve 12 datasets meticulously sourced from biomedical, and clinical domains as shown in Table 3.

#### Complex clinical case diagnosis task

we further assessed the effectiveness of Me-LLaMA in diagnosing complex clinical cases, a critical task given the increasing burden of diseases and the need for timely and accurate diagnosis to support clinicians. Recent studies demonstrate that LLMs have the potential to address this challenge.^39^ Specifically, we evaluated the diagnostic accuracy of Me-LLaMA on 70 challenging medical cases from the New England Journal of Medicine clinicopathologic conferences (NEJM CPCs) published between January 2021 and December 2022, as collected from an existing study.^39^ The NEJM CPCs are well-known for their unique and intricate clinical cases, which have long been used as benchmarks for evaluating challenging medical scenarios. In line with previous research,^39, 40^ we employed automatic evaluations based on top-K (where k = 1,2,3,4,5) accuracy, defined as the percentage of cases where the correct diagnosis appeared within the top-K positions of the differential diagnosis list predicted by the assessed models. We utilized GPT-4o, a state-of-the-art (SOTA) LLM, to automatically assess whether each diagnosis from the model’s differential diagnosis list matched the gold standard final diagnosis, consistent with these prior studies. Existing studies^40^ have shown that LLM-based automatic calculation of top-K accuracy is comparable to human evaluation. Besides automatic evaluation, we had a clinician specializing in internal medicine perform a manual evaluation of top-k accuracy (k = 1, 5). For more details on data processing, automatic evaluation, and human evaluation, see Appendix A.3.

### Evaluation Settings

We evaluated Me-LLaMA at two evaluation settings including zero-shot and supervised learning to evaluate their performance and generalization ability across various tasks compared to baseline models.

### Supervised Learning

In the supervised learning setting, we evaluated Me-LLaMA 13/70B base models’ performances adapted to downstream tasks. We conducted the task-specific finetuning on Me-LLaMA base models (Me-LLaMA task-specific) with each training set of assessed datasets in Table 6, and then assessed the performance of Me-LLaMA task-specific models on test datasets. We employed the AdamW optimizer. For datasets with fewer than 10,000 training samples, we fine-tuned the models for 5 epochs, while for larger datasets, the fine-tuning was conducted for 3 epochs. A uniform learning rate of 1e-5 was used across all datasets. Our baseline models including LLaMA2 Models (7B/13B/70B)^7^: they are open-sourced LLMs released by Meta AI. PMC-LLaMA 13B^2^ is a biomedical LLM continually pre-trained on biomedical papers and medical books. Meditron7B/70B^10^: they are medical LLMs based on LLaMA2-7B/70B, continual pre-trained with a mix of clinical guidelines, medical papers and abstracts.

#### Zero-shot Learning

We assessed our Me-LLaMA 13/70B-chat models’ zero-shot learning capabilities, which are key for new task understanding and response without specific prior training. We compared our models and baseline models’ zero-shot, using standardized prompts (detailed in Table A.2 shown in Appendix 0.2) for each test dataset from Table 2. We compared Me-LLaMA 13/70B-chat models with the following baseline models: ChatGPT/GPT-4^4,5^: SOTA commercialized LLMs. We used the version of “gpt-3.5-turbo-0301” for ChatGPT, and the version of “gpt-4-0314” for GPT-4. LLaMA2-7B/13B/70B-chat^7^ models were adaptations of the LLaMA2 series, optimized for dialogue and conversational scenarios. Medalpaca-7B/13B^3^ models were based on LLaMA-7B/13B, specifically fine-tuned for tasks in the medical domain. The PMC-LLaMA-13B-chat^2^ model is an instruction-tuned medical LLM based on PMC-LLaMA-13B. The AlpaCare-13B^13^ model is specifically tailored for clinical tasks based on LLaMA-2 13B by instruction tuning. Meditron 70B^10^ is a medical LLM, continually pre-trained with a mix of clinical guidelines, biomedical papers, and abstracts based on LLaMA2 70B.

## RESULTS

### Overall Performance: Medical Text Analysis

Table 4 compares the performance of our Me-LLaMA 13/70B foundation models against other open LLMs in the supervised setting. We can observe that the Me-LLaMA 13B model surpassed the similar-sized medical foundation model PMC-LLaMA 13B on 11 out of 12 datasets and outperformed the general foundation model LLaMA2 13B on 10 out of 12 datasets. Moreover, it is noticed that the Me-LLaMA 13B model was competitive with LLaMA2 70B and Meditron 70B, which have significantly larger parameter sizes, on 8 out of 12 datasets. As for 70B models, Me-LLaMA 70B achieved the best performance on 9 out of 12 datasets, when benchmarked against LLaMA2 70B and Meditron 70B.

Table 5 shows the zero-shot performance of Me-LLaMA chat models and other instruction tuned open LLMs with chat ability on various tasks. Among 13B models, Me-LLaMA 13B-chat outperformed LLaMA2 13B-chat, PMC-LLaMA-chat, Medalpaca 13B in almost all 12 datasets. Me-LLaMA outperformed AlpaCare-13B in 9 out of 12 datasets. Among models with 70B parameters, Me-LLaMA 70B-chat consistently outperformed LLaMA2-70B-chat on 11 out of 12 datasets. It is worth noting that Me-LLaMA13B-chat showed better performance than LLaMA2-70B-chat—a model with a significantly larger parameter size—on 6 out of 12 datasets and was competitive with the LLaMA2-70B-chat in 3 out of 6 remaining datasets.

**Figure 2:**
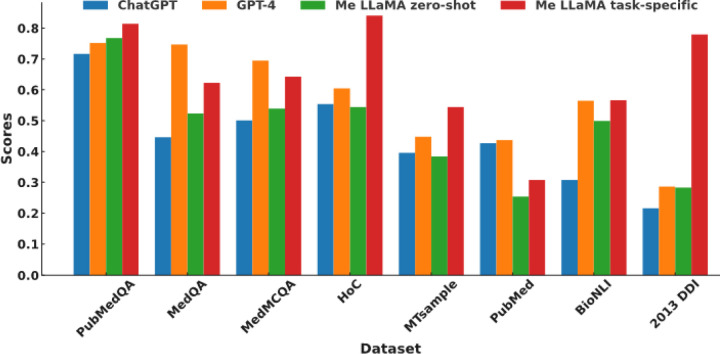
The performance comparison of Me-LLaMA models in both zero-shot (Me-LLaMA zero-shot) and supervised learning (Me-LLaMA task-specific) settings, against the zero-shot performance of ChatGPT and GPT-4.

Figure 2 further compares the performance of Me-LLaMA models in the zero-shot and supervised learning setting, against ChatGPT and GPT-4. Due to privacy concerns, which preclude the transmission of clinical datasets with patient information to ChatGPT and GPT-4, we conducted our comparison across 8 datasets that are not subject to these limitations. The results of ChatGPT and GPT-4 on three QA datasets are referenced from the OpenAI’s paper.^1^ We compared the Rouge-1^42^ score for the summarization dataset PubMed, the accuracy score for three QA datasets, and the Macro-F1 score for the remaining datasets. With task-specific supervised fine-tuning, Me-LLaMA models surpassed ChatGPT on 7 out of 8 datasets and excelled GPT-4 on 5 out of 8 datasets. In the zero-shot setting, Me-LLaMA models outperformed ChatGPT on 5 datasets; but it fell short on 7 datasets, when compared with GPT-4. It’s crucial to highlight that Me-LLaMA’s model size is significantly smaller—13/70B parameters versus at least 175B for ChatGPT and GPT-4. Despite this size discrepancy, Me-LLaMA models have showcased an impressive performance and a strong ability for supervised learning and zero-shot learning across a broad spectrum of medical tasks, underscoring its efficiency and potential in the field.

### Clinical Application: Complex Clinical Case Diagnosis

**Figure 3:**
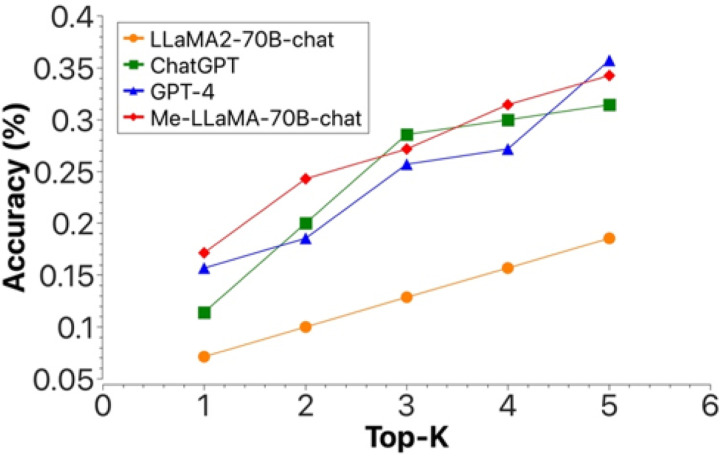
The top-k (1<=k<=5) accuracy of different LLMs in complex clinical case diagnosis, with automatic evaluation.

**Figure 4:**
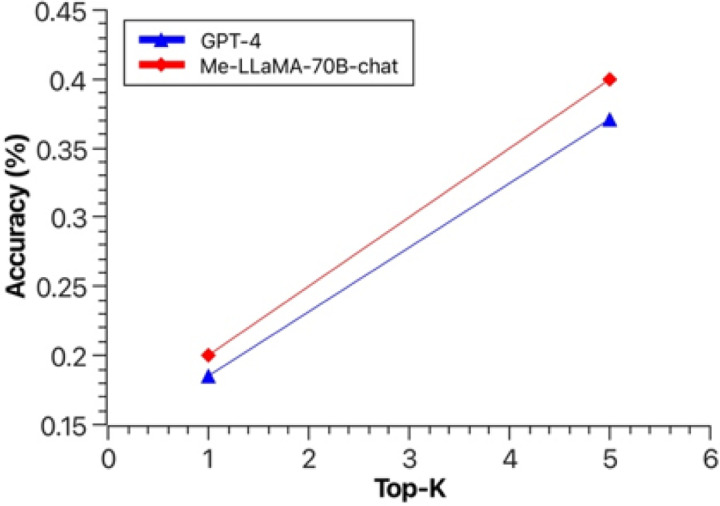
The top-1 and top-5 accuracy of Me-LLaMA-70B-chat and GPT-4 in complex clinical case diagnosis, with human evaluation.

Figure 3 shows the top-K (1 ≤ K ≤ 5) accuracy of Me-LLaMA-70B-chat, ChatGPT, GPT-4, and LLaMA2-70B-chat, in the complex clinical case diagnosis task. We can see Me-LLaMA-70B-chat model achieved comparable performance with GPT-4 and ChatGPT, and significantly outperforms LLaMA2-70B-chat. The human evaluation result in Fig. 4 again shows that Me-LLaMA-70B-chat outperformed GPT-4 in both top-1 and top-5 accuracy. These results demonstrated the potential of Me-LLaMA models for challenging clinical applications.

### Ablation Study: Impact of Continual Pretraining and Instruction Tuning

Table 6 compares the zero-shot performances of Me-LLaMA models and their backbone models LLaMA2, to illustrate the impact of continual pre-training and instruction tuning. Table 3 clearly demonstrates that both continual pre-training and instruction tuning significantly enhanced the zero-shot capabilities of models. For example, the Me-LLaMA 70B model showed an improvement in performance ranging from 2.1–55% across various datasets in comparison to the LLaMA2 13B model, highlighting the benefits of continual pre-training. The instruction tuning was also found to provide great increases in zero-shot performance. For instance, the Me-LLaMA-70B-chat model displayed enhancements in performance from 3.7–41.9% relative to the Me-LLaMA 70B foundation model, which had not undergone instruction tuning. This enhancement suggests the critical role of instruction finetuning in boosting the model’s ability to leverage context in learning tasks, even without supervised fine-tuning and prior examples.

## DISCUSSION

### Model Performance

We introduced a novel medical LLM family including, Me-LLaMA 13B and Me-LLaMA 70B, which encode comprehensive medical knowledge, along with their chat-optimized variants: Me-LLaMA-13/70B-chat, with strong zero-shot learning ability, for medical applications. These models were developed through the continual pre-training and instruction tuning of LLaMA2 models, using the largest and most comprehensive biomedical and clinical data. Compared to existing studies, we perform the most comprehensive evaluation, covering six critical text analysis tasks. Our evaluations reveal that Me-LLaMA models outperform existing open-source medical LLMs in various learning scenarios, showing less susceptibility to catastrophic forgetting and achieving competitive results against major commercial models including ChatGPT and GPT-4. Our work paves the way for more accurate, reliable, and comprehensive medical LLMs, and underscores the potential of LLMs on medical applications.

In the zero-shot setting, medical LLMs including GPT-4 displayed low performance on certain tasks, e.g., NER and RE, which are also noted by other studies.^43,44^ When compared with other NLP tasks with higher performance, we noticed that one of the main reasons for low performance is that LLMs’ responses often lacked the conciseness and precision expected, with instances of missing outputs noted. The unexpected outputs also cause significant challenges to automatic evaluation metrics. Therefore, more investigation is needed to further improve medical LLMs’ performance across tasks in the zero-shot setting^31^ and enhance the automatic assessment of these medical LLMs’ zero-shot capabilities. For the complex clinical case diagnosis, the Me-LLaMA-chat model had competitive performance and even outperformed GPT-4 in human evaluation. Existing studies have demonstrated GPT-4 is arguably one of the strongest LLMs in this task.^45^ The robust performance of Me-LLaMA showed potential in assisting challenging clinical applications. It is noticed that variations in test sizes and evaluation methods across different studies contribute to the observed differences in performance between GPT-4 in our paper and other studies. We also noted that both the Me-LLaMA-chat model and GPT-4 faced difficulties identifying the correct diagnosis within the top ranks, underscoring the difficulty of this task. Additionally, while the NEJM CPCs offer a rigorous test for these models, they do not encompass the full range of a physician’s duties or broader clinical competence. Therefore, complex clinical diagnosis remains a challenging area that demands more effective models and improved evaluation benchmarks to better capture the complexities of real-world clinical scenarios.

### Model Development

During our model development, we noticed the importance of diversity of the data sources during the pre-training and instruction-tuning phases. Our empirical results revealed that the PMC-LLaMA 13B model, which employed a data mix ratio of 19:1 between medical and general domain data, exhibited around 2.7% performance drop across both general and biomedical tasks. On the other hand, the Meditron models, 7B, and 70B, with a 99:1 mix ratio, demonstrated improvements in biomedical tasks, yet they still saw around 1% declines in the performance of general tasks. In contrast, our models, which adopt a 4:1 ratio, have shown enhancements in their performance for both general and medical tasks. This suggests that the integration of general domain data plays a vital role in mitigating the knowledge-forgetting issue during pre-training.^11,24,25^ However, determining the optimal balance between general domain data and specialized medical data is nontrivial, requiring careful empirical analysis. Future studies should examine methods to better determine the optimal ratio.

Our model development also underscores the balance between cost and effectiveness in pre-training versus instruction tuning of LLMs. Pre-training, exemplified by the LLaMA2 70B model, is notably resource-heavy, requiring about 700 hours on 160 A100 GPUs per epoch. Conversely, instruction tuning is far less resource-demanding, needing roughly 70 hours on 8 A100 GPUs per epoch, making it much more affordable than pre-training. Despite this, instruction tuning alone enhanced the performance of the Me-LLaMA-13B-chat model, achieving improvements ranging from 12% to 45% across 11 out of 12 datasets when compared to its backbone model – Me-LLaMA 13B, in the zero-shot setting. This efficiency advocates for prioritizing instruction tuning in scenarios with limited resources, highlighting its potential for cost-effective model enhancement.

### Use of Me-LLaMA Models

The Me-LLaMA models, available in both 13B and 70B sizes, as well as in base and chat-optimized versions, unlock a wide array of medical applications, guided by the crucial balance between model size and resource availability. The base models serve as robust foundations with extensive medical knowledge, adaptable through supervised fine-tuning for specialized tasks. Conversely, the chat versions excel in instruction-following ability and zero-shot learning, making them highly effective in zero-shot or few-shot learning scenarios. Larger models, like the 70B, provide deeper understanding and more complex reasoning abilities, ideal for comprehensive medical analyses. Yet, their deployment requires significant computing resources, posing challenges in resource-limited settings. On the other hand, the 13B models offer a practical compromise, balancing efficiency with effectiveness, thus ensuring broader accessibility for various applications. Our findings indicate that the Me-LLaMA 13B achieves performance on par with the 70B variant across most datasets, suggesting its viability for diverse medical tasks where computational or financial resources are a concern.

### Limitations

It is crucial to acknowledge the limitations of the current versions of Me-LLaMA models. Like all existing LLMs, they are susceptible to generating information with factual errors or biased information. To mitigate this, future studies could incorporate methodologies like reinforcement learning from human feedback (RLHF).^46^ This approach could align the models’ responses more closely with human values and ensure they are grounded in factual medical knowledge. Another limitation is the current token handling capacity, capped at 4096 tokens, which is a constraint inherited from the backbone LLaMA2 model. Addressing this limitation could involve extending the models’ capability to handle longer contexts. This could be achieved by integrating advanced attention techniques, such as sparse local attention,^47^ that are able to handle extensive contexts.

## Supplementary Material

Supplement 1

## Figures and Tables

**Table 1.** The comparison of Me-LLaMA models and existing open source medical LLMs.

Model	Backbone	Model size	Biomedical literature	Clinical notes	Continual pre-trainin (# of tokens)	Instruction tunin (# of Instructions)	Evaluation task	Release date
MedAlpaca	LLaMA	7/13B	✓	✗	-	160K	QA	04/14/2023
ChatDoctor	LLaMA2	7B	✓	✗	-	100K	QA	05/24/2023
AlpaCare	LLaMA	7/13B	✓	✗	-	52K	QA, Summarization	10/23/2023
Clinical LLaMA	LLaMA	7B	✗	✓	-	-	Classification	07/06/2023
Meditrom	LLaMA2	7/70B	✓	✗	48B	-	QA	11/27/2023
PMC-LLaMA	LLaMA	7/13B	✓	✗	79B	514K	QA	04/27/2023
**Me-LLaMA**	LLaMA2	13/70B	✓	✓	129B	214K	QA, NER, RE. Classification, Summarization, NLI, Medical Dianosis	06/05/2024

**Table 2:** The overall instruction tuning dataset.

Task	Type	Source		Size	Copy right
General	Conversation		Alpaca^19^	20,000	CC-BY-NC 4.0
			Dolly^20^		CC-BY-SA-3.0
			ShareGPT^21^		Apache-2.0
Biomedical	Conversation		HealthCareMagic^12^	20,000	Reserved by HealthCareMagic and Icliniq
			Icliniq^12^		
	Instructions		MedInstruct^13^	52,000	CC BY-NC 4.0
	Question Answering		Medical Flash Cards^3^	34,000	No commercialized use
			MEDIQA^22^	2,220	CC BY 4.0
			MedicationQA^23^	690	CC BY 4.0
			LiveQA^24^	634	CC BY 4.0
			WikiDocPatient^3^	5,490	CC BY-SA 4.0
			GuidelineQA	2,000	Common Crawl (other)
	Summarization		PubMed Central	10,000	CC BY
	Next Sentence Generation		PubMed Central	20,000	CC BY
	Key words prediction		PubMed Central	10,000	CC BY
	Causal Relation Detection		PubMed^25^	2,450	CC BY
	Relation Extraction		UMLS knowledge graph^2^	10,000	Openrail
Clinical	QA, summarization, classification, mortality prediction		MIMIC-III,^15^ MIMIC-IV16	30,000	PhysioNet credentialed health data use agreement 1.5.0

**Table 3:** Details of data splits and evaluation metrics of each dataset in the evaluation benchmark.

Datat		Task	Train		Valid	Test	Evaluation
PubMedQA* ^28^	QA		190,143	21,126		500	Accuracy, Macro-F1
MedQA^29^	QA		10,178	1,272		1,273	Accuracy, Macro-F1
MedMCQA* ^30^	QA		164,540	18,282		4,183	Accuracy, Macro-F1
EmrQA^31^	QA		122,326	30,581		26,804	Exact match, F1
i2b2^32^	NER		6,0875	7,400		7,451	Entity-level Macro-F1
DDI^33^	RE		18,779	7,244		5,761	Macro-F1
HoC^34^	Classification		1,108	157		315	Label-wise Macro-F1
MTSample^35^	Classification		4,999	500		999	Accuracy, Macro-F1
PubMed^36^	Summarization		117,108	6,631		6,658	Rouge, BERTScore
MIMIC-CXR^17^	Summarization		122,014	957		1,606	Rouge, BERTScore
BioNLI^37^	NLI		5,544	5,000		6,308	Accuracy, Macro-F1
MedNLI^38^	NLI		11,232	1,422		1,395	Accuracy, Macro-F1

**Table 4:** The supervised fine-tuning performance of various open source LLMs on six tasks.

Task		Dataset	Metric	LLaMA2 13B	PMC-LLaMA 13B	Me-LLaMA 13B	LLaMA2 70B	Meditron 70B	Me-LLaMA 70B
	PubMedQA		Acc	0.800	0.778	**0.802**	0.800	0.800*	**0.814**
Question answering			Macro-F1	0.560	0.544	**0.562**	0.560	-	**0.572**
	MedQA		Acc	0.467	0.456	**0.493**	0.598	0.607*	**0.623**
			Macro-F1	0.465	0.454	**0.487**	0.595	-	**0.621**
	MedMCQA		Acc	0.527	0.548	**0.557**	0.626	**0.651** *	0.643
			Macro-F1	0.524	0.545	**0.551**	0.625	-	**0.640**
	EmrQA		Acc	0.789	0.810	**0.857**	0.847	0.850	**0.854**
			F1	0.730	0.738	**0.751**	0.751	0.751	**0.751**
Named entity recognition	i2b2		Macro-F1	0.904	0.901	**0.906**	**0.913**	0.908	0.910
Relation extraction	DDI		Macro-F1	**0.622**	0.622	0.559	0.746	0.737	**0.779**
Classification	HoC		Macro-F1	**0.696**	0.422	0.684	0.818	0.702	**0.841**
	MTsample		Macro-F1	0.430	0.345	**0.451**	0.458	0.284	**0.544**
	PubMed		R-L	0.191	0.091	**0.197**	**0.211**	0.197	0.209
Summarization			BERTS	0.663	0.516	**0.679**	0.689	0.677	**0.700**
	MIMIC-CXR		R-L	0.437	0.139	**0.453**	0.440	0.458	**0.476**
			BERTS	0.816	0.694	**0.821**	0.813	0.824	**0.828**
Natural language inference	BioNLI		Macro-F1	0.409	0.332	**0.447**	0.447	0.444	**0.566**
	MedNLI		Macro-F1	0.881	0.868	**0.903**	0.884	0.897	**0.916**

* The performance of Meditron 70B on the PubMedQA, MedQA, and MedMCQA datasets is cited from the meditron paper^10^ to have a fair comparison.

**Table 5:** The zero-shot performance of various open source LLMs with chat capability.

**Task**	**Dataset**	**Metric**	**LLaMA2-13B-chat**	**PMC-LLaMA-chat**	**Medalpaca-13B**	**AlpaCare-13B**	**Me-LLaMA13B-chat**	**LLaMA2-70B-chat**	**Me-LLaMA70B-chat**
Question answering	PubMedQA	Accuracy	0.546	0.504	0.238	0.538	**0.700**	0.668	**0.768**
		Macro-F1	0.457	0.305	0.192	0.373	**0.504**	0.477	**0.557**
	MedQA	Accuracy	0.097	0.207	0.143	0.304	**0.427**	0.376	**0.523**
		Macro-F1	0.148	0.158	0.102	0.281	**0.422**	0.367	**0.521**
	MedMCQA	Accuracy	0.321	0.212	0.205	0.385	**0.449**	0.339	**0.539**
		Macro-F1	0.243	0.216	0.164	0.358	**0.440**	0.273	**0.538**
	EmrQA	Accuracy	0.001	**0.053**	0.000	0.001	0.048	0.050	**0.119**
		F1	0.098	0.304	0.040	0.198	**0.307**	0.251	**0.346**
Named entity recognition	i2b2	Macro-F1	0.143	0.091	0.000	**0.173**	0.166	0.321	**0.329**
Relation extraction	DDI	Macro-F1	0.090	0.147	0.058	0.110	**0.214**	0.087	**0.283**
Classification	HoC	Macro-F1	0.228	0.184	0.246	0.267	**0.335**	0.309	**0.544**
	MTsample	Macro-F1	0.133	0.083	0.003	**0.273**	0.229	0.254	**0.384**
	PubMed	Rouge-L	0.161	0.028	0.014	**0.167**	0.116	**0.192**	0.169
Summarization		BERTS*	0.671	0.128	0.117	**0.671**	0.445	**0.684**	0.678
	MIMIC-CXR	Rouge-L	0.144	0.139	0.010	0.134	**0.400**	0.131	**0.418**
		BERTS*	0.704	0.694	0.502	0.702	**0.797**	0.696	**0.787**
Natural language inference	BioNLI	Macro-F1	0.173	0.159	0.164	0.170	**0.195**	0.297	**0.436**
	MedNLI	Macro-F1	0.412	0.175	0.175	0.275	**0.472**	0.515	**0.675**

* BERTS: BERTScore.^41^

**Table 6:** The comparison of zero-shot performances among Me-LLaMA models and their backbone models LLaMA2.

Dataset	Metric	LLaMA2 13B (backbone)	Me-LLaMA13B (backbone + pre-train)	Me-LLaMA-13B-chat (backbone + pre-train + instruction tuning)	LLaMA270B (backbone)	Me-LLaMA70B (backbone + pre-train)	Me-LLaMA-70B-chat (backbone + pre-train + instructiontuning
PubMedQA	Acc	0.216	0.266	**0.700**	0.132	0.682	**0.768**
	Macro-F1	0.177	0.250	**0.504**	0.152	0.520	**0.557**
MedQA	Acc	0.000	0.000	**0.427**	0.005	0.281	**0.523**
	Macro-F1	0.000	0.000	**0.422**	0.009	0.350	**0.521**
MedMCQA	Acc	0.003	0.003	**0.449**	0.012	0.447	**0.539**
	Macro-F1	0.006	0.005	**0.440**	0.024	0.396	**0.538**
EmrQA	Acc	0.000	0.005	**0.048**	0.000	0.021	**0.119**
	F1	0.038	0.122	**0.307**	0.000	0.172	**0.346**
i2b2	Macro-F1	0.008	0.030	**0.263**	0.181	0.224	**0.329**
DDI	Macro-F1	0.035	0.036	**0.214**	0.034	0.118	**0.283**
HoC	Macro-F1	0.253	0.210	**0.335**	0.255	0.252	**0.544**
MTsample	Macro-F1	0.042	0.072	**0.229**	0.066	0.226	**0.384**
PubMed	R-L	**0.170**	0.168	0.116	0.167	0.119	**0.169**
	BERTS	**0.654**	0.654	0.445	0.654	0.654	**0.678**
MIMIC-CXR	R-L	0.051	0.172	**0.400**	0.059	0.137	**0.418**
	BERTS	0.566	0.697	**0.797**	0.577	0.649	**0.787**
BioNLI	Macro-F1	0.109	0.060	**0.195**	0.285	**0.449**	0.436
MedNLI	Macro-F1	0.172	0.206	**0.472**	0.265	0.256	**0.675**

## Data Availability

All datasets employed in the continual pre-training process and evaluation are accessible from their original published venues. The PubMed Central and PubMed Abstracts subset from The Pile are available at https://huggingface.co/datasets/EleutherAI/pile. MIMIC-IV and MIMIC-CXR datasets can be accessed under the PhysioNet Credentialed Health Data Use Agreement 1.5.0 at https://physionet.org/content/mimic-iv-note/2.2/ and https://physionet.org/ content/mimic-cxr/2.0.0/ respectively. The RedPajama data is open-released at https://huggingface.co/ datasets/togethercomputer/RedPajama-Data-1. Alpaca data is openly released at: https://github.com/tatsu-lab/stanford_alpaca. Dolly data is openly released at: https://huggingface.co/datasets/databricks/databricks-dolly-15k. Share GPT data can be accessed at: https://huggingface.co/datasets/anon8231489123/ShareGPT_Vicuna_unfiltered. The clinical instruction tuning data based on MIMIC-IV and MIMIC-CXR can be accessed under the PhysioNet Credentialed Health Data Use Agreement 1.5.0 through: https://huggingface.co/clinicalnlplab. The Medical Flash Cards and wikidoc QA datasets can be accessed at https://huggingface.co/medalpaca. Other remaining instruction tuning data can be openly accessed at: https://huggingface.co/clinicalnlplab. Me-LLaMA 13B and Me-LLaMA 70B models can be accessed at: https://physionet.org/content/me-llama/1.0.0/, subject to the completion of a credentialed health data use agreement.
