# Supplementary material for: Me-LLaMA: Medical Foundation Large Language Models for Comprehensive Text Analysis and Beyond
Source: Res Sq. 2024 Dec 18:rs.3.rs-5456223. Preprint. [Version 1] doi: 10.21203/rs.3.rs-5456223/v1 (PMC11702801; doi:10.21203/rs.3.rs-5456223/v1)
Supplement: Supplement 1 [file NIHPPRS5456223v1-supplement-1.pdf]

Supplementary Files

This is a list of supplementary files associated with this preprint. Click to download.

- APPENDIX.docx
